# Application value of transparent-cap-assisted colonoscopy: A randomized clinical trial

**DOI:** 10.1097/MD.0000000000043885

**Published:** 2025-08-15

**Authors:** Qiong Wu, Zhenguo Qiao, Fang Wang, Lihua Xu, Xin Ling, Mingxia Xia, Jian Zhao

**Affiliations:** a Department of Gastroenterology, Suzhou Ninth People’s Hospital, Suzhou Ninth Hospital Affiliated to Soochow University, Suzhou, China; b Department of Gastroenterology, Gaochun People’s Hospital of Nanjing, Nanjing, China; c Department of Gastroenterology, Affiliated Nantong Hospital of Shanghai University (The Sixth People’s Hospital of Nantong), Nantong, China; d Department of Emergency, Suzhou Ninth People’s Hospital, Suzhou Ninth Hospital Affiliated to Soochow University, Suzhou, China.

**Keywords:** cap-assisted colonoscopy, cecal intubation, polyp/adenoma detection rate, standard colonoscopy

## Abstract

Colonoscopy provides limited protection against interval colorectal cancer. This study aims to investigate the application value of transparent-cap-assisted colonoscopy (CAC) regarding cecal intubation time and polyp detection rate. This prospective randomized controlled trial recruited 480 patients in our hospitals. The patients were randomly assigned to either standard colonoscopy (SC) or CAC procedures. Six attending physicians or above conducted the colonoscopies. Caecal intubation time and polyp detection rate were compared between the 2 groups. No significant differences in epidemical characteristics were noted between the 2 groups. When compared to the SC group, CAC had no significant difference in terms of cecal intubation rate, and polyp/adenoma detection rate, but had significant differences in terms of ileal intubation rate, cecal time, total colonoscopy time, and when compared to SC, CAC is a secure, practical, and simple technique that improves ileal intubation rate, and cecal time, and offers a higher polyps/adenomas detection rate in subjects aged ≥50 years. This study is a basic research and does not include complex cases. In our In our next trials, this simple method will be compared to determine whether it is possible to enhance protection against interstitial colon cancer for difficult colonoscopies (including those subjects aged ≥50 years with polyps, postoperative intestinal adhesions, constipation or obesity, etc).

## 1. Introduction

**Figure 1.**
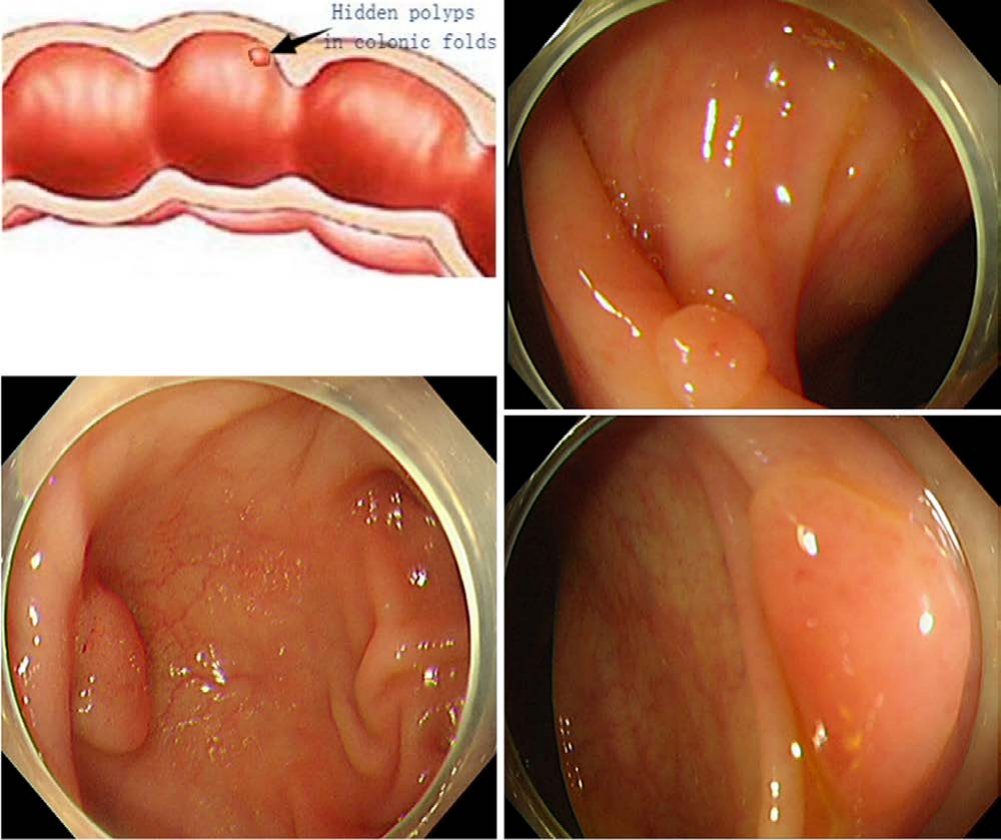
Hidden polyps in the colonic folds.

Colorectal cancer (CRC) is the fourth leading cause of cancer-related mortality and colorectal adenomas are the precursors of colon tumors. Colorectal cancer incidence and mortality rates can be reduced by detecting and removing polyps/adenomas via colonoscopy.^[1,2]^ Several studies, however, have shown that the prevention of colorectal cancer by colonoscopy is related to the location (proximal colon vs distal colon), and its prevention effect on proximal colon cancer needs to be further improved.^[3]^ Colonoscopy is a complex procedure that requires sophisticated equipment and expertise. Technical indicators including the failure rate of caecal intubation and the rate of missed polyps are used to assess the quality of colonoscopy.^[4,5]^ Short cecal intubation times are advantageous because: they require less anesthetic medication, colonic inflation results in less discomfort, allow adequate withdrawal time for accurate examination. Several strategies including chromoendoscopy, wide angle colonoscopy, narrow band imaging, fluorescence confocal endomicroscopy, etc, have been evaluated to improve the detection rate of polyps/adenomas during colonoscopy.^[6,7]^ For instance, cap-assisted colonoscopy (CAC) is 1 promising technique. A transparent cap is a simple plastic device attached to the top of a colonoscopy before a colonoscopy. However, several trials have reported conflicting results regarding transparent caps improving caecal intubation time and polyp detection rate^[6,8]^ (Fig. 1). This study sought to compare CAC with standard colonoscopy (SC) for cecal intubation time and detection rate of polyp/adenoma.

## 2. Materials and methods

### 2.1. Patients

A prospective randomized controlled trial comparing SC and CAC was conducted in our hospitals between June 2019 and October 2021. A total of 480 consecutive subjects scheduled for their first ever colonoscopy as a routine health check were enrolled. All were aged 18 years or above. Exclusion criteria included previous colonic resection, colon cancer, inflammatory bowel disease, ischemic colitis, acute gastrointestinal bleeding, polyposis syndrome, use of antiplatelet or anticoagulant agents, poor general condition, pregnancy or breast feeding, or inability to provide informed consent.^[9,10]^ Patient epidemical data were recorded. This study was approved by the ethics committee of the sixth people’s Hospital of Nantong, Nantong, and Suzhou Ninth People’s Hospital, Suzhou, Jiangsu Province, China.

All patients completed the study protocol. Baseline demographics of subjects were similar between the 2 groups, including age, gender, BMI, indication, bowel preparation quality, and sedation medication doses (*P* > .05, Table 1).

**Table 1:** Baseline demographics of all patients n (%).

	CAC (n = 240)	SC (n = 240)	*X*^2^/T	*P* value
Gender			0.21	.86
Male (%)	118 (49.2)	115 (47.9)		
Female (%)	122 (50.8)	125 (52.1)		
Age (yr)*	51.6 ± 15.4	53.6 ± 14.8	0.57	.50
Subjects aged (n, %)			0.24	.63
<50 yr	73 (30.4)	78 (32.5)		
≥50 yr	167 (69.6)	162 (67.5)		
Body mass index (kg/m^2^)*				
<50 yr	24.1 ± 4.4	24.7 ± 4.8	1.86	.08
≥50 yr	23.6 ± 4.5	24.6 ± 5.2	1.91	.06
Indications (n, %)			0.12	.93
Abdominal pain	47 (19.6)	45 (18.7)		
Abdominal distention	36 (15.0)	33 (13.8)		
Diarrhea	52 (21.7)	57 (23.7)		
Constipation	18 (7.5)	16 (6.7)		
Hemafecia	32 (13.3)	31 (12.9)		
CRC screening	55 (22.9)	58 (24.2)		
Bowel preparation			0.14	.94
Good	117 (48.8)	126 (52.5)		
Satisfactory	95 (39.6)	87 (36.3)		
Poor	28 (11.6)	27 (11.2)		

CAC = cap-assisted colonoscopy, CRC = colorectal cancer, SC = standard colonoscopy.

* mean ± SD.

After enrollment, the patients were randomly assigned to the SC and CAC groups by opening an opaque sealed envelope containing the program assignments, patients with inadequate colon preparation or inability to touch the cecum were excluded. Patients were blinded to the allocation. The routine bowel preparation included a clear liquid diet a day before colonoscopy, and fasting a day before the examination. All patients began to drink 4 L of polyethylene glycol electrolyte lavage solution at a rate of 250 mL every 15 minutes before colonoscopy 7 hours.^[9]^ Colonoscopies were conducted by 6 attending physicians or senior physicians, each of them had performed more than 10,000 colonoscopies. All procedures were performed under conscious or deep sedation with a combination of intravenous fentanyl (Nhwa Pharma Co., Ltd., Xuzhou, China), and propofol (Fresofol 1%; LIBANG Pharma Co., Ltd., Xi`an, China) administered by the assistant or attending anesthetist. Boston Gut Readiness Score was used in the study.

The transparent plastic cap (D-14304; Olympus Optical Corp., Toyko, Japan) was fitted and fixed 4 mm beyond the tip of the colonoscope (PCF- Q 260JI; Olympus Optical Co., Tokyo, Japan). Although the rim of the cap was visible on the monitor, the visual field was not limited as the endoscopist was able to see through the transparent cap.^[11]^

### 2.2. Colonoscopy procedure

During and after the colonoscopy, data on operation times, polyps, complications, and other parameters were collected. The procedure was considered successful if the colonoscope reached the cecum, which was identified by either the appendicular orifice or ileocecal valve landmarks. In all cases, terminal ileal intubation was attempted and successful intubation was recorded.

Polyps/adenomas were removed in a standard manner and sent for pathological examination. If a small polyp was discovered during the insertion and the endoscopist decided to remove it, the time spent would be deducted from the insertion time, but included in the total procedure time.

### 2.3. The results of the measurement

The caecal intubation time was the primary endpoint, whereas the polyp detection rate was the secondary endpoint. Other endpoints included ileal intubation rate, total colonoscopy time, and complication rate. All complications (including bleeding or perforation) were documented. The endoscopists were asked to document any technical difficulties encountered while using the cap, including cap loss, fluid inhalation, mucosal cleaning, or therapeutic intervention.

### 2.4. Statistical analysis

Statistical analysis was performed using SPSS version 22.0 (SPSS, Chicago). Student *t* test was used to assess differences between the 2 groups for normally distributed continuous variables; the χ^2^ or Fisher’s exact test was used for categorical variables. Statistical significance was defined as *P* < .05 (2-tailed).

## 3. Results

### 3.1. Cecal intubation rate

Overall cecal intubation rate was 97.1% (466/480), and the terminal ileum intubation rate was 86.9% (417/480). The cecal intubation rate showed no significant difference between CAC (238/240, 99.2%) and SC (228/240, 95.0%; *X*^2^ = 0.11, *P* > .05), but the ileal intubation rate showed significant difference between CAC (225/240, 93.8%) and SC (172/240, 71.7%; *X*^2^ = 3.89, *P *< .05; Table 2).

**Table 2:** Primary outcomes: performance of colonoscopy n (%).

	CAC	SC	*X*^2^/T	*P* value
Cecal intubation rate	238/240 (99.2)	228/240 (95.0)	0.11	.94
Ileal intubation rate	225/240 (93.8)	172/240 (71.7)	3.89	.04
Cecal time (mean ± SD)	9.3 ± 7.1	10.3 ± 6.8	1.98	.04
Total colonoscopy time (mean ± SD)	15.5 ± 9.5	18.6 ± 8.4	2.05	.03

Note: The unit of Cecal time and Total colonoscopy time is min and the number of the sample is 387.

CAC = cap-assisted colonoscopy, SC = standard colonoscopy.

### 3.2. Cecal intubation time

The mean insertion time to reach the cecum was shorter in the CAC group than that in the SC group, and the difference was statistically significant (9.3 ± 7.1 minutes vs 10.3 ± 6.8 minutes, *P* = .04). Mean total colonoscopy times were 15.5 ± 9.5 5 minutes in the CAC group and 18.6 ± 8.4 in the SC group (*P* = .03; Table 2). The mean withdrawal time (5.95 ± 1.58 minutes in CAC vs 5.98 ± 1.45 minutes for SC, *P* = .75).

### 3.3. Polyp detection rate

Among the 480 subjects, the total number of polyps detected by CAC was higher than that of SC, and the difference was statistically significant (145 vs 106, *P* = .04); Similarly, the proportion of patients with polyps in the CAC group was higher than that in the SC group, but the difference was not statistically significant (39.2% vs 29.2%; *P* = .06). For subjects aged 50 years and above, 49.7% of polyps were detected by CAC, and 32.7% of polyps by SC, which was different between the 2 groups (*P* = .04; Table 3).

**Table 3:** Secondary outcomes: polyp detection rate n (%).

	CAC (n = 240)	SC (n = 240)	*X* ^2^	*P* value
Total number of polyps	145	106	3.99	.04
Total number of polyps with size ≤5 mm	95	66	3.92	.04
Subjects with polyps (%)	94 (39.2)	70 (29.2)	2.62	.06
Subjects aged ≥50 yr with polyps (%)	83/167 (49.7)	53/162 (32.7)	4.08	.04

CAC = cap-assisted colonoscopy, SC = standard colonoscopy.

### 3.4. Complications and adverse events

The use of the cap did not cause any complications. Only one patient had transparent cap displacement during colonoscopy, and the cap had to be removed by colonoscopy again. CAC suspected hemorrhoids in 2 cases of anal pain.

## 4. Discussion

Colonoscopy is commonly used to diagnose lower digestive tract diseases and screen for colorectal polyp/adenoma.^[2,3]^ Colon polypectomy can prevent 76% to 90% of the expected CRC, however, the presence of interval CRCs (CRCs diagnosed between the time of a negative screening colonoscopy and that of the next recommended colonoscopy) is an important question, possible causes of these intervals cancers include colonoscopy failure or undiscovered lesions.^[12]^ Therefore, improving the efficacy of colonoscopy is extremely important. A series of large colonoscopy studies have shown that up to 10% of cases did not reach the cecum, with the missed rate of polyp/adenoma by colonoscopy being 17% to 48%. Improved colonoscopy techniques (standardized withdrawal time,^[13–15]^ retroflection of the colonoscope in the right colon, quality of bowel preparation^[16,17]^) and advanced colonoscopy equipment (third eye retroscope, color endoscopy, wide angle colonoscopy, high-definition magnifying endoscopy, narrow band imaging, fluorescence confocal endoscopy, etc), have made significant progress in improving colonoscopic results.^[18,19]^ However, beginners find it difficult to master the complex endoscopic technology, and community hospitals find it difficult to promote expensive equipment. Furthermore, incomplete resection of serrated polyps may affect the development of interval colon cancer due to its subtle appearance.

The main advantage of CAC is its low cost, simple-to-use, and secure technology. The cap, when attached to the top end of the colonoscope, helps in flattening the colonic folds and locating the colon cavity in the view of the endoscopist (Fig. 1). The lateral side mucosa can be examined through the transparent wall of the cap during insertion and withdrawal,^[20]^ and the blind area of the fold is easily observed because it can be straightened to improve the view, hence the celiac cavity can often be seen. CAC is an effective rescue method for caecal intubation failure, the device can shorten the time of caecal intubation and improve the detection of colonic polyps for inexperienced colonoscopists, contributing to early detection and resection of colorectal lesions against colorectal cancer development.^[19,20]^

Herein, the overall cecal intubation rate was 97.1% (466/480), CAC had shorter cecal intubation times than SC (*P* < .05), CAC had significantly higher detection rates for polyps than SC (*P* < .05). No significant difference in the proportion of subjects with at least one polyp was noted between CAC (94/240, 39.2%) and SC (70/240, 29.2%; *P* > .05), despite the proportion of CAC being higher than SC, consistent with the results of Tee HP,^[10]^ Tang Z^[14]^ and inconsistent with the results of Pohl H,^[6]^ Desai M.^[7]^ Extremely low air insufflations with the use of CAC cause less bowel inflation and generate fewer bowel angulations, thus improving cecal intubation. Since the known polyp miss rate is approximately 24%,^[2]^ the question of any benefit of CAC has not been adequately answered and there is a need for further randomized controlled trials in the future. The difference between CAC and SC in detecting serrated polyps is also necessary.

This study has several limitations. First, the study is a 2-center study. Secondly, the results may not be generalizable to the general population. Thirdly, as the endoscopic examination went very smoothly, the withdrawal time of a few subjects did not exceed 6 minutes. Fourthly, the endoscopists could not be blinded to CAC compared to SC. Finally, fecal matter sticking to the cap may impair the view and take time to clean.

In conclusion, we conducted a randomized controlled study of CAC and SC and discovered that experienced endoscopists are quick and effective with and without transparent caps. Attending physicians and above endoscopists found no significant differences in the results between CAC and SC in the population aged under 50 years. However, statistical differences were evident between CAC and SC in ileum intubation rate, cecum time and polyp detection rate in individuals aged over 50 years. CAC is a secure, practical, simple to use method for improving production efficiency. This study is a basic research and does not include complex cases. Future research is necessary to determine whether transparent caps are beneficial in ADR. In our In our next trials, this simple method will be compared to determine whether it is possible to enhance protection against interstitial colon cancer for difficult colonoscopies (including those subjects aged ≥50 years with polyps, postoperative intestinal adhesions, constipation or obesity, etc). Then, in our subsequent research, CAC will be compared with chromoendoscopy, or magnifying endoscopy in patients with difficult colonoscopy to find out who could detect more polyps or early-stage cancers. Furthermore, additional studies are necessary to determine whether there are specific subgroups of patients who may benefit more from the use of a cap, whether to train endoscopists in the use of the device, and appropriate training methods.

## Author contributions

**Conceptualization:** Zhenguo Qiao.

**Data curation:** Lihua Xu.

**Formal analysis:** Xin Ling.

**Funding acquisition:** Lihua Xu, Mingxia Xia.

**Methodology:** Jian Zhao.

**Software:** Jian Zhao.

**Supervision:** Zhenguo Qiao.

**Validation:** Zhenguo Qiao.

**Writing – original draft:** Qiong Wu, Fang Wang.

**Writing – review & editing:** Jian Zhao.
